# *Nipponnemertes incainca* sp. n. Adoption of the new taxonomic proposal for nemerteans (Nemertea, Cratenemertidae)

**DOI:** 10.3897/zookeys.693.12015

**Published:** 2017-08-22

**Authors:** Jaime Gonzalez-Cueto, Lyda R. Castro, Sigmer Quiroga

**Affiliations:** 1 Grupo de Investigación MIKU, Facultad de Ciencias Básicas, Universidad del Magdalena, Carrera 32 No 22 – 08, Santa Marta D.T.C.H. Magdalena, Colombia. 470004; 2 Grupo de Investigación GIESEMOL, Facultad de Ciencias Básicas, Universidad del Magdalena, Carrera 32 No 22 - 08, Santa Marta D.T.C.H. Magdalena, Colombia. 470004

**Keywords:** New species, Nemertea, COI, Caribbean coast of Colombia

## Abstract

A new species Nipponemertes incainca is described from the intertidal zone of Santa Marta, Colombia. A new recent approach based on both morphological and molecular characters is applied for the description. The main characteristics of the species are: red color, head shield-shaped with a mid-dorsal cephalic ridge, furrows pre-cerebral inconspicuous with few faint ridges orthogonal to furrow axis, two irregular groups of eyespots situated at lateral margins in precerebral cephalic region, proboscis provided with papillae and 12 nerves, stylet smooth supported on an oval basis, and two pouches containing 3–4 accessory stylets each. The sequence of the COI gene was analyzed as an additional support for the new species.

## Introduction

Nemerteans (phylum Nemertea), commonly known as ribbon worms or Rhynchocoela, comprise a cosmopolitan group of bilateral, coelomate, and unsegmented worms (Turbeville 2002). The major synapomorphy supporting the monophyly of the phylum is the presence of an eversible proboscis housed in a fluid-filled cavity, the rhynchocoel (*ibid*). Around 1,300 species of nemerteans are recognized, most of which are found in marine environments; nevertheless, freshwater and terrestrial species are also known (Gibson 1995, Kajihara et al. 2007). Among nemerteans the genus *Nipponnemertes* contains 18 species of marine benthic worms; the interwoven muscular layers in the rhynchocoel wall, and large cerebral sense organs extending behind the brain are the two main traits that distinguish them from most other monostiliferan genera (Friedrich 1968).

Of the 36 species of nemerteans documented for the Caribbean Sea (Corrêa 1961, 1963, Kirsteuer 1973, 1974, 1977, Schwartz and Norenburg 2005, Gonzalez-Cueto et al. 2014), 13 have been reported to be present on the Colombian coast (Kirsteuer 1977, Gonzalez-Cueto et al. 2014). However, the majority of these species have been recorded from a single locality (Santa Marta Bay) despite nemerteans being an abundant component of the macrofauna communities in Colombia (Dueñas 1998, Trujillo et al. 2009). Taxonomic studies on nemerteans from Colombia are scarce. Species identifications traditionally require a detailed study of the internal anatomy, which is considered to be difficult and time consuming. This is because several morphological characters are doubtful, subjective, poorly defined, and plastic, in addition to a lack of taxon experts (Sundberg et al. 2016a).

Sundberg et al. (2016a) highlight these problems for the taxonomy of nemerteans and, to advance the study of nemerteans, they suggest transitioning from a traditional, difficult, and often unreliable taxonomy to a more integrative process of describing species based on external morphological characteristics that are easily observable combined with molecular data. Together this would facilitate more accurate species identifications, even for the non-specialist.

Herein, the method proposed by Sundberg et al. (2016a) is used to describe a new species of ribbon worm from Colombia. The external appearance of the worm and photos of the histological section of the proboscis are presented, in addition to a molecular analysis using the mitochondrial gene COI. The COI sequence of one specimen is deposited in GenBank and whole specimens fixed in formalin and other tissue pieces preserved in absolute alcohol are deposited in the “Centro de Colecciones Biológicas de la Universidad del Magdalena” for future molecular and morphological studies.

## Materials and methods

Four specimens were hand-collected on the rocky littoral from Inca-Inca Bay, Santa Marta, Colombia (11°12'30.2"N; 74°13'54.5"W). Individuals were relaxed in 7% MgCl_2_ solution isotonic to seawater and photographed “*in vivo*” with a digital camera Nikon D7100 with a 60 mm ED Micro-Nikkon lens. Details of morphological characters were photographed with a stereomicroscope Leica M205A with an integrated Camera Leica DFC450. Detailed images of the proboscis and stylets were obtained by pressing the specimens between a slide and a coverslip (obligating them to protrude the proboscis) and photographing them with a microscope Zeiss Axiolab A1 with an integrated camera Zeiss ERc5s. Two specimens were fixed in 100% ETOH for molecular purposes and two in 10% formalin for future morphological analysis.

Two additional specimens previously collected and deposited in the “Centro de Colecciones Biológicas de la Universidad del Magdalena, CBUMAG” (Gonzalez-Cueto et al. 2014) were also examined. Cross sections of the proboscis were obtained from one these specimens (CBUMAG:NEM: 0049). For that, the proboscis was embedded in paraffin; sectioned at 7µm thickness with an AO 820 Spencer microtome, and stained with H&E. Coverslips were mounted with Permount®.

Total DNA was extracted from one entire worm fixed in 100% ETOH, using the DNeasy Blood & Tissue^®^ Kit following the manufacturer’s protocol (Qiagen, Valencia, CA, USA). The partial COI gene was amplified with universal primers described in Folmer et al. (1994). The PCR was performed with 2 μL template in a 25 μL volume with final concentrations of 2 mM MgCl_2_, 5X buffer PCR (no MgCl_2_ BIOLINE^®^), 0.4 μM of each primer, 0.4 μM of each dNTP, and 2 units Taq (BIOLASETM, BIOLINE^®^). The PCR conditions were: 1 min at 95 °C, followed by 35 cycles of 15 s at 95 °C, 1 min at 40 °C, 1.5 min at 72 °C, and there was a final extension period of 5 min at 72 °C. The sequence was edited with ProSeq (Filatov 2009) and aligned with all the sequences from *Nipponnemertes* accessible in GenBank using the ClustalW algorithm available in MEGA (Tamura et al. 2011) with default parameters. Following the barcoding approach suggested by Hebert et al. (2003), a matrix of intraspecific and interspecific evolutionary genetic distances was made using the Kimura’s two parameter model K2P (Kimura 1980), also available in Mega (Tamura et al. 2011).

## Results

### Taxonomy

#### Family: Cratenemertidae Friedrich, 1968

##### Genus: *Nipponnemertes* Friedrich, 1968

###### Nipponnemertes incainca sp. n.

Taxon classificationAnimaliaMonostiliferaCratenemertidae

http://zoobank.org/942EBF8B-976E-4952-B2C5-FCFD32BF690D

Fig. 1 A–F

####### Material examined.

Holotype: COLOMBIA Santa Marta, Rodadero Inca-Inca beach (11°12'30.2"N, 74°13'54.5"W), intertidal zone under boulders, whole specimen in 70% ethanol (CBUMAG:NEM: 0056). Total body length 18.5 mm, 1 mm wide.

Paratypes: COLOMBIA Santa Marta, Taganga (11°15'51.23"N, 74°11'31.54"W), intertidal zone under boulders covered by sponges, whole specimen in 70% ethanol (CBUMAG:NEM: 0043). Total body length 11.7 mm, 1.8 mm wide.

COLOMBIA Santa Marta, Rodadero Inca-Inca beach (11°12'30.2"N, 74°13'54.5"W), intertidal zone under boulders, transverse histological sections of the proboscis; rest of specimen in 70% ethanol (CBUMAG:NEM:0049). Total body length 22.5 mm, 2.05 mm wide.

COLOMBIA Santa Marta, Rodadero Inca-Inca beach (11°12'30.2"N, 74°13'54.5"W), intertidal zone under boulders; tissue in absolute ethanol (CBUMAG:NEM:00068, CBUMAG:NEM:00069).

An entire additional worm, collected in Inca-Inca beach (11°12'30.2"N, 74°13'54.5"W) was used for DNA extraction. Sequence data for 615 bp of Cytochrome C Oxidase Subunit I deposited in GenBank under accession number KX879856 (see alignments with other congeners in supplemental information).

**Figure 1.**
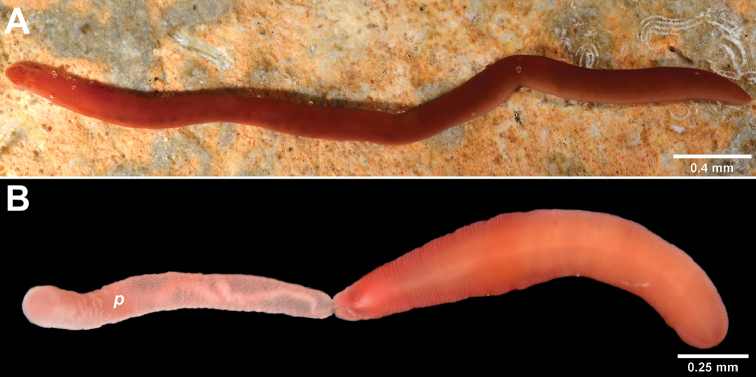
*Nipponnemertes incainca* sp. n. **A** Dorsal view of entire worm **B** Ventral view of entire worm. Abbreviation: *p* proboscis

####### Etymology.

The specific epithet refers to the “Inca-Inca beach” site from which most of the specimens were collected; this name is in apposition.

####### Diagnosis.

*Nipponnemertes incainca* sp. n., like other members of *Nipponnemertes*, has a mid-dorsal cephalic ridge, is capable of retracting the head into the body when disturbed, and is capable of swimming. However, in this new species the anterior furrows and their secondary transverse grooves are faintly visible both macro- and microscopically and they are not visible in a ventral view.

####### Description.

**Figure 2.**
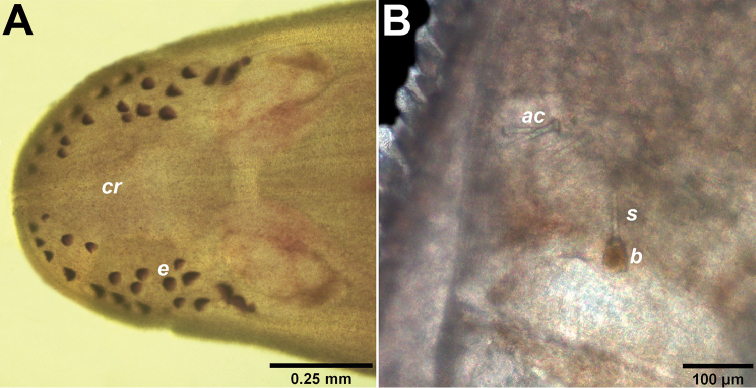
*Nipponnemertes incainca* sp. n. **A** Detail of ocelli **B** Microscopic detail of stylet and accessory stylets. Abbreviations: *cr* cephalic ridge, *e* eyespot, *s* central stylet, *b* base of stylet, *ac* accessory stylets.

**Figure 3.**
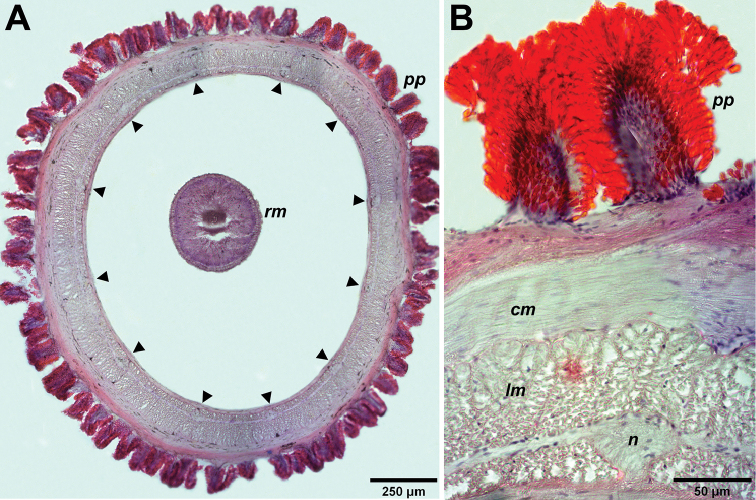
*Nipponnemertes incainca* sp. n. **A** Transverse sections of the proboscis; nerves are highlighted by arrowheads **B** Microscopic detail of transverse section showing the proboscis papillae. Abbreviations: *pp* proboscis papillae, *lm* longitudinal muscles, *cm* circular muscles, *rm* retractor muscles of the proboscis, *n* nerve.

Relaxed length from 11.7 mm to 22.5 mm and width 1 to 2 mm. Dorsal side uniformly bright red color (Fig. 1A). Ventral side lighter than dorsal side (Fig. 1). Head shield-shaped, slightly demarcated from rest of body but without V-shape cephalic groove and not wider than trunk. Mid-dorsal cephalic ridge present in head (Fig. 1A, 2A). Frontal organ with small cirrus. Cerebral organ furrows pre-cerebral, inconspicuous, with few faint ridges orthogonal to furrow axis. Brain distinguishable as a pale brown bilobed structure through dorsal and ventral body wall. Two irregular groups of eyespots situated at lateral margins in precerebral cephalic region (Fig. 2A), extending beyond brain parallel to lateral nerve cords. Rhynchopore subterminal. Proboscis long and stout, with papillae (Fig. 3B), pink in color when everted (Fig. 1B). Stylet (length: 87.4 µm) smooth, supported on an oval basis (54 × 38.3 µm); two pouches containing 3-4 accessory stylets each (Fig. 2B). Twelve proboscidial nerves present (Fig. 3A–B). This species was found among sponges and brown algae underneath rocks, and in the crust formed by sediment inside the crevices of rocks in the littoral zone. Worms capable of swimming with strong undulating movements.

####### Diferential diagnosis.

We compared morphological characters of *Nipponnemertes incainca* sp. n. with the 18 valid species of the genus, according to Gibson (1995) and Kajihara et al. (2007) (Table 1).

The most similar species in color, arrangement of ocelli and numbers of proboscidial nerves to *Nipponnemertes incainca* sp. n. is *N. pulchra* and it might easily represent an intraspecific variation. However, in the intraspecific and interspecific genetic distance matrix (table 2), the interspecific distance between *N. incainca* sp. n. and *N. pulchra* was 21.03%, which exceeds the highest limits given by Sundberg et al. (2016b) for the Hoplonemertea. Therefore, the fact that the new species lacks the V-shape structure formed by the cephalic grooves, and the accessory stylet in the basis of the central armature, present in *N. pulchra*, is enough to discriminate the two species.

**Table 1.** Remarks about morphological and behavioral traits useful to discriminate the species of the genus *Nipponnemertes*. Reference after authority in species column.

Species	Body Coloration,	Number of Proboscis Nerves	Mid-dorsal cephalic ridge	Shape and distinctness of posterior dorsal V-shaped cephalic groove	Other Noteworthy Characters
*Nipponnemertes incainca* sp. n.	Solid bright red color pattern without designs	12	Present	Lacking	Anterior furrows and secondary transverse grooves present, but faintly visible both macro and microscopically. Inhabits rocky littoral zone
*Nipponnemertes africanus* (Wheeler, 1940) Berg, 1985	“*White, pink, pinkish yellow or buff, lighter anteriorly and deepest on back*” (Berg 1985). Mottled and dotted with white gonads	11	Present (McDermott 1998)	Present. Two posterior dorsal cephalic grooves, V-shaped but not joined medially (McDermott 1998, P. 252)	Faint head-glands, open close to external opening of rhynchodeum and disappear just before brain. Found between roots of alga *Hypnea specifica*, low on shore
*Nipponnemertes arenaria* (Uschakov, 1927) Chernyshev 1993	Margins of body lighter in color				Inhabits muddy sand (Chernyshev 1993)
*Nipponnemertes bimaculatus* (Coe, 1901) Gibson & Crandall, 1989	Head flesh in color; rest of body is deep red, brownish red, or brownish orange; lighter on ventral surface. Possesses pair scalene triangle-shaped cephalic marks and a narrow longitudinal line of dark color on dorsal surface of esophageal region	14 or 16	Present (Coe 1905, plate 18)	Lacking	Central stylet very long and slender, mounted on a remarkably tiny base
*Nipponemertes danae* (Friedrich, 1957) Friedrich, 1968	Dorsal surface red, ventral white; color description based on Coe’s description of *Nipponnemertes drepanophoroides* (Coe 1905, p. 282)				Original description is vague and lacks important information. According to Berg (1985) it is synonym of *N. pulchra*
*Nipponnemertes drepanophoroides* (Griffin, 1898) Friedrich, 1968	Red above, white beneath				Lacks intestinal caeca
*Nipponnemertes fernaldi* Iwata, 2001	Pale brown on dorsal surface and darker on the ventral side (colorless lateral margins)	14	Present	Oblique, limited to dorsal surface	
*Nipponnemertes madagascarensis* (Kirsteuer, 1965) Friedrich, 1968	Ochre on dorsal surface, stained with irregular reddish-brown blotches	9	Lacking	Lacking	
*Nipponnemertes magnus* (Punnett, 1903) Berg, 1985	Light orange-brown	20			
*Nipponnemertes marioni* (Hubrecht, 1887) Berg, 1985	“*Dorsally blue-green, yellow-green, pale buff or light brown, and ventrally pale buff light orange-brown*” (Berg 1985)	15			
*Nipponnemertes occidentalis* (Coe, 1905) Friedrich, 1968	Blotchy dark reddish brown or pale ground color throughout whole dorsal surface, and “*ventral surface without color*” (Coe 1905)				Highly developed intestinal caecum. Caecal appendage in esophagus and one in stomach
*Nipponnemertes ogumai* (Yamaoka, 1947) Crandall et al., 2001	Uniformly orange (Kajihara et al. 2014) although originally described as bright vermilion (Crandall et al. 2001)	16	Present (Kajihara et al. 2014)	Present, but not significantly developed	Minute ocelli gathered as a triangle on each side of head
*Nipponnemertes pacificus* (Coe ,1905) Friedrich, 1968	Reddish or brownish dorsal surface, pale beneath	14	Lacking	Lacking	Cerebral sense organs remarkably large and highly specialized. Highly developed esophageal caecum (Coe 1905)
*Nipponnemertes pulchra* (Johnston, 1837) Berg, 1972	“*Dorsal surface varying between brown, red and pink. Lateral parts of body and ventral surface always much lighter, longitudinal dorsal swelling on head often somewhat darker*” (Berg 1985)	8-14 (normally 12)	Present	Dorsally, clearly marked and darker than rest of body. Does not reach midline on ventral surface	Presence of accessory stylet in basis of central armature. This character has been highlighted as one of best criteria to recognize *N. pulchra*
*Nipponnemertes punctatulus* (Coe, 1905) Friedrich, 1968	Pale brown or yellowish white with numerous darker brown spots on dorsum and white ventrum (head white with two dark blotches). Proboscis transparent, with pinkish stylet basis (Iwata 2008)	15	Present	Lacking	Iwata (2008) recorded 12, 13 or 16 proboscis nerves in worms collected in United States
*Nipponnemertes rubella* (Coe, 1905) Crandall & Norenburg, 1999	Deep flesh color, pale orange, or pale red; much paler and usually grayish beneath	14			Great development of body parenchyma and intestinal caeca
*Nipponnemertes sanguinea* Riser, 1998	Dorsum buffy white to pale yellow to orange with reddish lines (aggregation of red blood corpuscles in blood vessels), ventral side paler, brain lobes pink	12	“*Not evident*” (Riser 1998)	Lacking	Presence of red blood corpuscles
*Nipponnemertes schollaerti* (Wheeler, 1934) Berg, 1985	Pale buff color	14	Lacking (Wheeler 1934, p. 265)	Lacking	
*Nipponnemertes variabilis* (Korotkevich, 1983) Chernyshev, 1993	Beige dorsal and ventrally	12-13	Lacking	Separating strongly head from rest of body	

**Table 2.** COI-based matrix of interspecific and intraspecific genetic distances, using Kimura’s two-parameter model K2P (Kimura 1980). GenBank accession numbers: *Nipponnemertes incainca* sp. n. (KX879856); *N. bimaculatus* (AJ436909); *N. pulchra* (KP697761- KP697767); *N. punctatulus* (AJ436910); *N. ogumai* (AB920907); *Nipponnemertes* sp. 1 (HQ848598); *Nipponnemertes* sp. 2 (HQ848599); *Nipponnemertes* sp. 3 (KU230295).

	*Nipponnemertes incainca* sp. n.	*Nipponemertes bimaculatus*	*Nipponnemertes pulchra*	*Nipponnemertes punctatulus*	*Nipponnemertes ogumai*	*Nipponnemertes* sp. 1	*Nipponnemertes* sp. 2	*Nipponnemertes* sp. 3
*Nipponnemertes incainca* sp. n.	×	×	×	×	×	×	×	×
*Nipponemertes bimaculatus*	15.62	×	×	×	×	×	×	×
*Nipponnemertes pulchra*	21.03	20.12	0.09	×	×	×	×	×
*Nipponnemertes punctatulus*	17.13	8.61	18.39	×	×	×	×	×
*Nipponnemertes ogumai*	56.60	53.33	48.44	55.31	×	×	×	×
*Nipponnemertes* sp. 1	21.44	19.14	4.50	18.31	52.26	×	×	×
*Nipponnemertes* sp. 2	16.00	18.82	10.32	18.91	47.30	10.92	×	×
*Nipponnemertes* sp. 3	17.13	8.61	18.39	0.00	55.31	18.31	21.00	×

## Discussion

Approximately, 2.2 million (σ 0.18) species inhabit the marine ecosystems, yet 91% of these still await description (Tittensor et al. 2010; Mora et al. 2011). The rate at which these species become extinct has reached an unprecedented degree that is much higher than the rate of new species discovered (Dirzo and Raven 2003; Scheffers et al. 2012). The new taxonomic approach of Sundberg et al. (2016a) might help facilitate the description of new species of nemerteans, which otherwise would be underestimated or overlooked. With this approach some morphological characters and molecular data of the new species will be available to scientists in order to have a more integrative assessment of biodiversity. However this approach should be interpreted cautiously because some species, such as the one described here, might require the revision of some internal features (*i.e*. the number of nerves in the proboscis).

*Nipponnemertes incainca* sp. n. was recorded as Cratenemertidae sp. by Gonzalez-Cueto et al. (2014) and probably it is also the same species recorded as Cratenemertidae spp. by Collin et al. (2005) from “Bocas del Toro (Panama)”. Misidentification of nemerteans is common in the environmental assessments of marine ecosystem around the world (Sundberg et al. 2016a). In fact, in Colombia, many specimens remain named as Nemertea sp. even in biological collections such as the “*Museo de Historia Natural Marina de Colombia (INVEMAR)*” and the “*Centro de Colecciones Biológicas de la Universidad del Magdalena*”. The standardization of the taxonomic and behavior-based character matrix proposed by Sundberg et al. (2016a), applied in this survey (Table 3), and the use of molecular markers (e.g. COI) increase the value of taxonomic identifications in the future. Our study expands the known number of nemertean species of the Caribbean coast of Colombia from 12 to 13. In addition, it encourages a new generation of taxonomists to begin or to continue working on this neglected group of animals.

**Table 3.** Character checklist. List of external characters that could be checked in order to provide a species description with comparable characters. Modified from Sundberg et al. (2016a).

	Character	Character state	Code
1.	Biology	Free-living	0
2.	Habitat	Marine	0
3.	Benthic divisions	Littoral	1
5.	Habitat	Epibenthic	2
6.	Substratum	Rock/boulders	3
7.	Behavior when mechanically disturbed	Contracts without coiling into a spiral	0
	External morphology		
8.	Cephalic furrows/slits	One pair	1
9.	Distribution of anterior cephalic furrows/slits	Dorsal	1
10.	Shape of anterior (dorsal) cephalic furrows (viewed with tip of head directing forwards)	Ventral transversal	2
12.	Head clearly demarcated from body	Head not wider than trunk	2
13.	Position of cephalic furrows	If single pair in front of brain lobes	1
14.	Shape of head/cephalic lobe	Shield-shaped	10
15.	Head viewed laterally	Without extensions	0
16.	Cross section shape of body	Rounded cylindrical	0
17.	Shape of posterior tip	Bluntly rounded	3
18.	Eyes	Eyes arranged in lateral rows or groups on each side of head	7
19.	Eye distinctiveness	Eyes visible from ventral side	0
20.	Eye morphology	Simple	0
21.	Relative eye size	All eyes more or less of equal size	0
22.	Eye position relative to brain lobes	Confined principally or entirely to precerebral cephalic region but may extend back to above brain	0
23.	General body color	No obvious color	0
24.	Primary dorsal body color	Red	0
25.	Color pattern	Absent	0
26.	Color of blood	Red	0
27.	Proboscis armature	With central and accessory stylets	2
28.	Number of accessory stylet pouches	Two	0
29.	Number of stylets in each accessory stylet pouch	Three or four	1
30.	Stylet : basis/stylet ratio	1.5:1	1
31.	Stylet shaft	Smooth and straight	0
32.	Shape of stylet basis	Oval (rounded)	0
33.	Median waist of stylet basis	Absent	0
34.	Proboscis used for locomotion	Yes	1
35.	Proboscis pore	Subterminal, ventral	1
38.	Lateral margins	No distinction in color	1
39.	Distribution of bristles/cirri	Only on head	1

## Supplementary Material

XML Treatment for Nipponnemertes incainca

## Supplementary materials

Supplementary material 1 COI partial gene alignments of all *Nipponnemertes* sequences from GenBank Data type: molecular data Explanation note: Fasta format This dataset is made available under the Open Database License (http://opendatacommons.org/licenses/odbl/1.0/). The Open Database License (ODbL) is a license agreement intended to allow users to freely share, modify, and use this Dataset while maintaining this same freedom for others, provided that the original source and author(s) are credited.Jaime Gonzalez-Cueto, Lyda R. Castro, Sigmer Quiroga
